# Late‐onset Krabbe disease presenting as spastic paraplegia – implications of GCase and CTSB/D

**DOI:** 10.1002/acn3.52078

**Published:** 2024-06-04

**Authors:** Rebecca Mächtel, Jan‐Philipp Dobert, Ute Hehr, Alexander Weiss, Matthias Kettwig, Lucia Laugwitz, Samuel Groeschel, Manuel Schmidt, Philipp Arnold, Martin Regensburger, Friederike Zunke

**Affiliations:** ^1^ Department of Molecular Neurology University Hospital Erlangen, Friedrich‐Alexander‐Universität Erlangen‐Nürnberg (FAU) Erlangen Germany; ^2^ Center for Human Genetics Regensburg Germany; ^3^ Department of Pediatrics and Pediatric Neurology University Medical Center Göttingen, Georg August University Göttingen Göttingen Germany; ^4^ Department of Pediatric Neurology University Children's Hospital Tübingen Tübingen Germany; ^5^ Department of Neuroradiology FAU Erlangen Germany; ^6^ Institute of Functional and Clinical Anatomy FAU Erlangen Germany; ^7^ Department of Stem Cell Biology FAU Erlangen Germany; ^8^ Deutsches Zentrum Immuntherapie (DZI) University Hospital Erlangen Erlangen Germany

**Keywords:** cathepsin B, cathepsin D, enzymatic activity, Krabbe disease, late‐onset, β‐glucocerebrosidase

## Abstract

**Objective:**

Krabbe disease (KD) is a multisystem neurodegenerative disorder with severe disability and premature death, mostly with an infancy/childhood onset. In rare cases of late‐onset phenotypes, symptoms are often milder and difficult to diagnose. We here present a translational approach combining diagnostic and biochemical analyses of a male patient with a progressive gait disorder starting at the age of 44 years, with a final diagnosis of late‐onset KD (LOKD).

**Methods:**

Additionally to cerebral MRI, protein structural analyses of the β‐galactocerebrosidase protein (GALC) were performed. Moreover, expression, lysosomal localization, and activities of β‐glucocerebrosidase (GCase), cathepsin B (CTSB), and cathepsin D (CTSD) were analyzed in leukocytes, fibroblasts, and lysosomes of fibroblasts.

**Results:**

Exome sequencing revealed biallelic likely pathogenic variants: *GALC* exons 11–17: 33 kb deletion; exon 4: missense variant (c.334A>G, p.Thr112Ala). We detected a reduced GALC activity in leukocytes and fibroblasts. While histological KD phenotypes were absent in fibroblasts, they showed a significantly decreased activities of GCase, CTSB, and CTSD in lysosomal fractions, while expression levels were unaffected.

**Interpretation:**

The presented LOKD case underlines the age‐dependent appearance of a mildly pathogenic *GALC* variant and its interplay with other lysosomal proteins. As GALC malfunction results in reduced ceramide levels, we assume this to be causative for the here described decrease in CTSB and CTSD activity, potentially leading to diminished GCase activity. Hence, we emphasize the importance of a functional interplay between the lysosomal enzymes GALC, CTSB, CTSD, and GCase, as well as between their substrates, and propose their conjoined contribution in KD pathology.

## Introduction

Globoid cell leukodystrophy or Krabbe disease (KD) [MIM * 245200] is a rare autosomal recessive neurodegenerative disorder occurring in approx. 1 to 100,000–400,000 births.^1^, ^2^ The diagnosis of KD can be challenging due to the broad spectrum of clinical features and highly variable disease onset. To date, more than 340 variants in the *GALC* gene, encoding the lysosomal enzyme β‐galactocerebrosidase (GALC), have been identified for KD^3^. While the majority of cases is characterized by an onset before 6 months of age (early‐infantile KD) and a fatal outcome within 2 years, also later onset during infancy (late‐infantile KD) with a similarly rapid disease course are known.^4^ Furthermore, with the availability of enzymatic and genetic testing, the phenotypic spectrum was widened, as the diagnosis of adolescent/adult onset cases remains undetected less often.^5^ These late‐onset KD (LOKD) cases may present with only slight developmental regression and/or motor impairment including lower limb spasticity or reduced fine motor skills.^6^ Some patients may present as late as within the fourth decade with initial clinical features resembling spastic paraparesis.^7^ Interestingly, there is only a limited genotype–phenotype correlation. Early‐infantile cases are often caused by loss of function variants and consequently exhibit severely diminished GALC activity, leading to generalized neuronal demyelination and overall globoid cell pathology.^8^ Adolescent/adult onset cases, in turn, were associated with a highly variable residual enzyme activity and a variable age at onset as late as in the seventh decade.^6^ Hematopoietic stem cell transplantation is the only therapeutic option to date, but only performed in presymptomatic or early stages of the disease course.^8^

**Figure 1:**
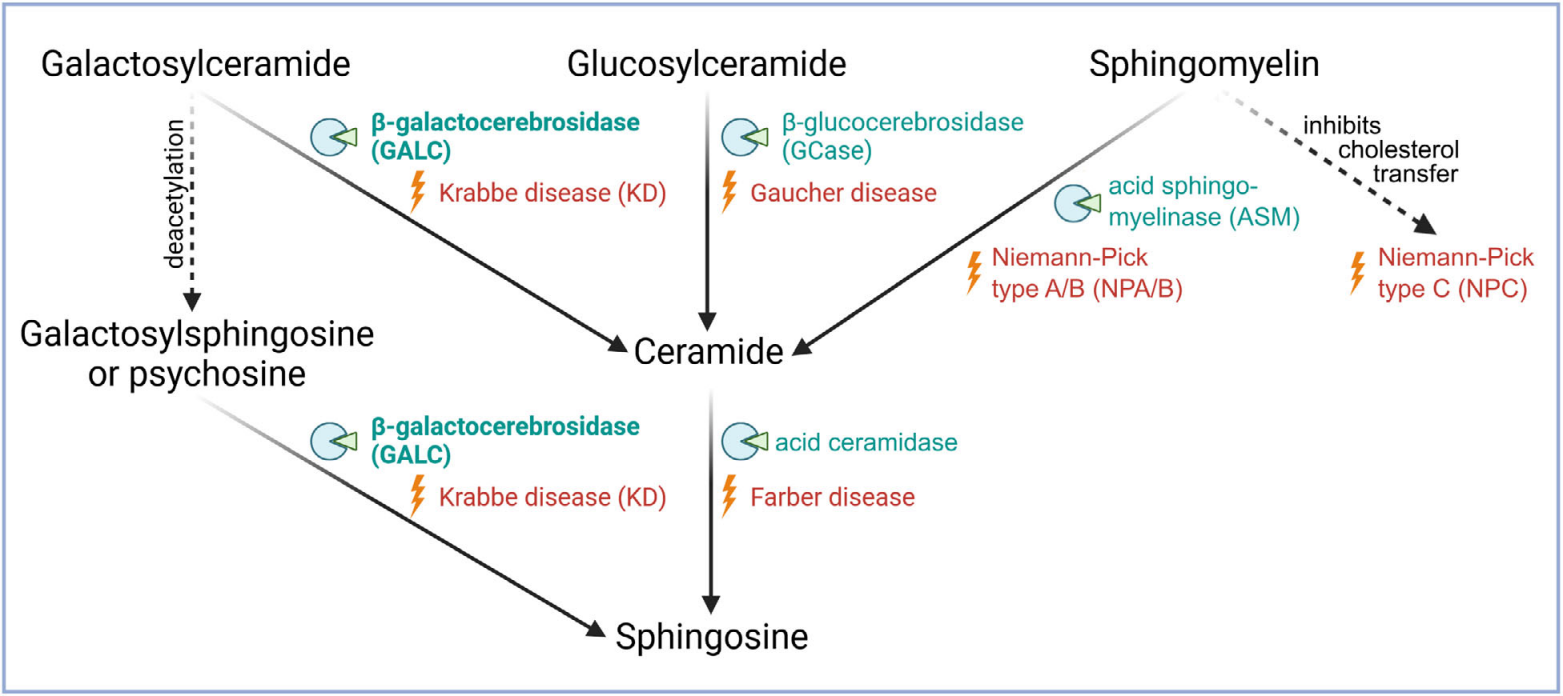
Schematic overview of glycosphingolipid metabolism and associated enzymes and diseases. Galactosylceramide, glucosylceramide, and sphingomyelin are metabolized to ceramide (and the respective carbohydrate residue (not shown)) by different lysosomal enzymes (green). Dysfunction or lack of each enzyme can lead to specific lysosomal storage disorders (LSDs, orange) with severe neurodegeneration effects. Deficiencies in β‐galactocerebrosidase (GALC) can also cause accumulation of cytotoxic galactosylsphingosin (psychosine). All named diseases are related to impaired sphingolipid (i.e., ceramide) metabolism, thus being classified as sphingolipidoses. See text for more details. *Figure created with Inkscape and BioRender.com*.

GALC is a glycoside hydrolase that catalyzes the hydrolysis of galactosylceramide to β‐galactose and ceramide, and of galactosylsphingosine (psychosine) to β‐galactose and sphingosine^9^, ^10^, ^11^ (Fig. 1). Dysfunctional GALC or reduced enzymatic activity leads to accumulation of nondegraded galactosylceramide and cytotoxic psychosine within the lysosome.^12^, ^13^ Hence, KD belongs to the group of lysosomal storage disorders (LSDs). The lysosomal storage phenotype of KD is accompanied by demyelination in the central and peripheral nervous system, but also apoptotic and inflammatory responses contribute to KD development and progression. Common clinical hallmarks of a neuropathological KD diagnosis are gigantic polynuclear glia cells, also termed globoid cells, white matter demyelination, and signs of axonal and neuronal degeneration.^14^, ^15^, ^16^, ^17^, ^18^

Functional overlap of GALC is seen with the lysosomal enzymes β‐glucocerebrosidase (GCase) and acid sphingomyelinase (ASM), as they all metabolize their substrates (glucosylceramide as well as sphingomyelin) to ceramide (Fig. 1). Dysfunction of any of the three enzymes leads to a lack of ceramide and impairment of neuronal cell function.^19^, ^20^, ^21^ Pathogenic variants of *GBA1* (encoding GCase) are known to cause Gaucher's disease, the most common LSD, and have also been described as one of the highest genetic risk factors for the development of the neurodegenerative disorder Parkinson's disease (PD).^22^ Deficiency of ASM, caused by mutations in the *SMPD1* gene, leads to Niemann–Pick diseases types A or B (NPA/B), as sphingomyelin accumulates in the lysosomes of neurons, liver, spleen, and other organs.^20^ Importantly, recent genome‐wide association studies also associated *GALC* with PD among other genes encoding lysosomal enzymes such as *CTSB* and *CTSD*.^23^, ^24^, ^25^, ^26^, ^27^

In this study, we clinically characterize a case of LOKD carrying a deletion and a missense *GALC* variant in compound heterozygous state. Additionally, we perform biochemical and cellular analyses to evaluate the interplay of GALC with other components of the lysosomal pathway in order to gain a better understanding of the molecular disease mechanisms.

## Materials and Methods

### Subjects

The index patient and respective controls (Table 1) were recruited from the Movement Disorders Outpatient Unit of the Department of Molecular Neurology, University Hospital Erlangen, after providing written informed consent according to the local Institutional Review Board approval (“Biobank to analyze biomarkers and generate human cellular models of neurological diseases,” No. 17‐259‐B, Ethics Committee of the Friedrich‐Alexander‐Universität Erlangen‐Nürnberg, Erlangen, Germany).

**Table 1:** Clinical characteristics of the patient and matched controls.

Line	C1	C2	C3	LOKD (patient)
Identifier	UKERf1E4–1	UKERf7MN‐1	UKERfO3H‐1	UKERf3H3–1
Sex	Male	Male	Male	Male
Family history	Negative for neurological disorders	Negative for neurological disorders	Negative for neurological disorders	Negative for neurological disorders
Age at motor onset	–	–	–	44 years
Age at biopsy	53 years	52 years	71 years	56 years
Spastic Paraplegia Rating Scale (SPRS) at biopsy	–	–	–	6
Presenting symptom	–	–	–	Stumbling

### Gene sequencing

Genomic DNA was prepared from peripheral blood, processed by whole‐exome Nextera enrichment™ and high throughput sequencing (Illumina™ NextSeq500), open clinical exome screen using NxClinical 5.2 (© BioDiscovery, Inc), mapping to ref genome hg19, bioinformatic sequence assessment for sequence variants and copy number variations in a virtual panel: variant calling and annotation with SeqNext (JSI medical systems GmbH) of the coding regions and flanking splice sites of *ATL1, CYP7B1, KIF5A, REEP1, SPAST, SPG7, SPG11, ZFYVE26*, and *GALC*.

### Tissue culture

Human primary fibroblasts (hereafter referred to as fibroblasts only) were derived from skin biopsies as described previously.^28^ They were cultured in T75 flasks in DMEM with 15% v/v FBS, 1x penicillin/streptomycin, and 2 ng/mL FGF (R&D Systems, United States, #233‐FB). Twice a week, each line was either passaged when confluent or the culture medium was replaced with fresh prewarmed medium. Cells were analyzed before reaching their 10th passage.

### Sample preparation and lysosome enrichment of fibroblasts

Cell pellets were lysed in 150 μL Triton buffer (1% v/v Triton X‐100, 10% v/v glycerol, 150 mM NaCl, 25 mM HEPES at pH 7.4) containing 1x protease inhibitor cocktail (cOmplete Protease Inhibitor Cocktail, Roche, Switzerland, #11836145001), 50 mM NaF, 2 mM NaVO_4_, and 1 mM phenylmethylsulphonyl fluoride (PMSF). After incubation on ice for 30 min, samples were treated with a tip sonicator (2× 10 s and 1× 20 s; 1 s on/1 s off; 60% amplitude) and spun for 20 min at 20,000 × *g*, 4°C. Protein concentrations of the supernatant were determined using a Micro BCA™ Protein‐Assay‐Kit (ThermoFisher Scientific Inc., United States, #23235). For activity assays, cell pellets were lysed in the according lysis buffer (without protease inhibitors, see below) instead of Triton buffer. For lysosome enrichment, cells were homogenized in 400 μL sucrose hepes buffer (0.25 M sucrose, 10 mM HEPES, 0.1 M EDTA at pH 7.4) by using a teflon pestle and a conical glass vessel (Glas‐Col, Terre Haute, IN; #099D GT31). After spinning at 6800 × *g*, 5 min, 4°C, the supernatant was transferred into a new tube and the process repeated with the pellet an another 200 μL of buffer. Both supernatants were combined and after spinning at 17,000 × *g*, 10 min, 4°C; the pellet was used for cell lysis as explained above.

### Western blot analyses

For whole‐cell lysates: Approx. 20 μg of proteins were loaded onto 8–10% SDS‐PAGES and separated by electrophoresis at 120–140 V for 1–1.5 h. Proteins were then transferred onto nitrocellulose or polyvinylidene fluoride (PVDF) membrane (Amersham Protran Premium 0.2 μm NC, Cytiva, and Millipore Immobilon‐FL PVDF 0.45 μm, Merck KGaA, respectively) and the Trans‐Blot Turbo Transfer System (Bio‐Rad Laboratories, Inc.), according to manufacturer instructions. The membranes were blocked with 2% w/v fish protein in 0.1% v/v TBST‐T for 1 h. Primary antibodies were diluted 1:1,000 in 0.1% TBS‐T and incubated over night at 4°C. After washing with 0.1% TBS‐T, the second antibody (1:10,000 in 0.1% TBS‐T) was incubated with the membrane for 2 h at room temperature and again washed with 0.1% TBS‐T. Detection of the fluorescent antibodies was done using the iBright FL1500 Imaging System (ThermoFisher Scientific Inc.) and normalized to β‐actin as loading control.

For lysosome‐enriched cell lysates: Approx. 15 μg of proteins were loaded onto 4–12% Bolt™ Bis‐Tris Plus Mini Protein Gels (Thermo Fisher Scientific Inc., # NW04125BOX) and separated by electrophoresis at 120 V for 1–1.5 h. Proteins were then transferred onto polyvinylidene fluoride (PVDF) membrane (as above) at a constant voltage (30 V) for 1:10 h. The membranes were blocked with Intercept® (TBS) blocking buffer (Li‐Cor, Lincoln, NE; #927–60001) for 1 h. Antibody incubation as above, yet diluted in Intercept® T20 (TBS) Antibody Diluent (#927–65001). Detection of the fluorescent antibodies was done using Odyssey (LI‐COR Biosciences, Lincoln, NE) imaging system and normalized to Coomassie Brilliant Blue (CBB) of the blotted protein gel.

**The following antibodies were used for western blotting:**

LAMP‐2A Polyclonal antibody (AMC2) (rabbit), Thermo Fisher Scientific Inc./Invitrogen (#51–2200)
LIMP2 Polyclonal Antibody (goat), Thermo Fisher Scientific Inc./Invitrogen (#PA5‐19111).
Anti‐P62 antibody (mouse), abcam (#ab56416)
Anti‐Glucocerebrosidase antibody, C‐terminal (rabbit), Sigma Aldrich (#G4171)
Anti‐Cathepsin B antibody (goat), R&D Systems (#AF953)
Anti‐Cathepsin D antibody (mouse), BD Transduction Laboratories™ (#610800)
Anti‐beta‐Actin antibody (mouse), abcam (#ab5441)
Human/Mouse/Rat GAPDH Antibody, R&D Systems (#AF5718)
Donkey anti‐Mouse IgG (H + L) Highly Cross‐Adsorbed Secondary Antibody, Alexa Fluor™ 680, ThermoFisher Scientific Inc. (#A10038)
Donkey anti‐Rabbit IgG (H + L) Highly Cross‐Adsorbed Secondary Antibody, Alexa Fluor™ 680, ThermoFisher Scientific Inc. (#A10043)
Donkey anti‐Rabbit IgG Secondary Antibody, IRDye® 800CW, LI‐COR Biosciences GmbH (#926–322193)
Donkey anti‐Goat IgG Secondary Antibody, IRDye® 800CW, LI‐COR Biosciences GmbH (#926–32214)
Donkey anti‐Goat IgG (H + L) Cross‐Adsorbed Secondary Antibody, Alexa Fluor™ 488, ThermoFisher Scientific Inc. (#A11055)

### GALC activity assays

GALC activity was assessed following the previously described protocol.^29^ β‐galactocerebrosidase activity was measured as an internal reference. Contrary to the original publication, we are aware that the fluorescent assay utilizing the substrate 6‐Hexadecanoylamino‐4‐methylumbelliferyl‐beta‐D‐galactopyranoside (HMGal) exhibits lower sensitivity compared to the radioactively labeled substrate method. Therefore, enzyme activity of GALC was determined in peripheral white blood cells and skin fibroblasts using both a fluorometry‐based method and the radioactively labeled substrate method, following previously established procedures.^30^

### CTSB, CTSD, and GCase protein activity assays

For all activity assays, 5 μg total protein were used per replicate (samples and negative controls) in a total sample volume of 100 μL. For CTSB and CTSD activity levels, samples were incubated in lysis buffer (0.2% v/v Triton X‐100, 50 mM sodiumacetate, 0.1 M NaCl, 1 mM EDTA) at 37°C for 30 (CTSB) and 60 min (CTSD), in the presence of either 20 μM CTSB substrate (Enzo Life Science Z‐RR‐AMC Cathepsin substrate BML‐P137‐0010) or 10 μM CTSD substrate (Enzo Life Sciences Cathepsin D & E substrate (fluorogenic), #BML‐P145‐0001) and 25 μM Leupeptin (Enzo Life Science Leupeptin (synthetic) Cathepsin B inhibitor, # ALX‐260‐009‐M005) (for CTSD only). Negative controls contained 75 μM Leupeptin (CTSB) or 200 μg/mL Pepstatin A Protease Inhibitor (ThermoFisher Scientific Inc., #78436).

For GCase activity levels, samples were incubated at 37°C for 60 min in 100 μL 4MU activity buffer (McIlvaine buffer pH 5.4 (0.2 M Na_2_HPO_4_, 0.1 M citric acid), 0.25% v/v Triton X‐100, 0.25% w/v sodium taurocholate), containing 1 mM 4MU substrate solution (4‐Methylumbelliferyl β‐D‐glucopyranoside, Sigma Aldrich, #M3633). After incubation, 100 μL of stop solution (0.5 M glycine in NaOH, pH 10.4) was added. Negative controls contained buffer instead of substrate.

Activities were quantified in black 96‐well‐plates (Nunc MaxiSorp, ThermoFisher Scientific Inc., #437111) with a microliter plate reader (Molecular Devices LLC., SpectraMax Gemini, United States; excitation/emission wavelengths: 356/440 nm for CTSB, 322/381 nm for CTSD, 365/445 nm for GCase) and corrected by the corresponding blank (buffer only) value.

### GCase activity assays in leukocytes

#### Radioactive GCase activity assay

The assays used in the present study specifically evaluate the lysosomal GCase enzyme activity by measuring the degradation capacity of glucocerebroside. The lysosomal GCase activity of the index case LOKD was assessed in blood leukocytes via radioactive measurement using the natural substrate (glucocerebroside [Stearoyl‐1‐14C]) following previously reported procedures.^31^, ^32^

#### Fluorometric GCase activity assay

The lysosomal GCase activity of the index case LOKD was assessed in blood leukocytes via fluorometric measurement using the artificial substrate (4‐Methylumbelliferyl β‐D‐glucopyranoside) following previously reported procedures.^33^, ^34^

### Lysosomal live cell GCase and CTSB activity assays in fibroblasts

Lysosome‐specific GCase activity of living cells was measured via the fluorescent probe (5‐(Pentafluorobenzoylamino)Fluorescein Di‐β‐D‐Glucopyranoside) (PFB‐Gluc; ThermoFisher Scientific Inc., #P11947), CTSB activity via Magic Red® Cathepsin‐B Assay Kit (Immunochemistry Technologies, United States, #937). The experimental procedure was adapted from Cuddy and Mazzulli.^35^ Human fibroblasts were seeded at 100,000 cells per well on a black 96‐well plate with transparent bottom (Corning, United States, #3603) in fibroblast medium. For each line, eight wells were seeded. After 2 days, 200 nM bafilomycin 1A (Baf, Santa Cruz Biotechnology, United States, #sc‐201550A) was added to four of the eight seeded wells per line for 1 h to inhibit lysosomal enzyme activity (control wells), while the remaining wells equivalently received DMSO only (sample wells). Subsequently, the medium in all wells was changed to 50 μL of fresh medium containing 100 μg/mL of PFB‐Gluc, 1x Magic Red substrate (sample wells) or PFB‐Gluc/Magic Red substrate and Baf (control wells). After 1 h of incubation with the fluorogenic probes, all wells were aspirated, washed once with medium, and filled with 100 μL of Fluorobrite™ DMEM (Gibco, United States, #A1896701) + 15% v/v FCS (+ Baf for control wells). The first fluorescence measurement was taken (PFB‐Gluc: λ_ex_: 485 nm; λ_em_: 538 nm; cutoff: 495 nm; Magic Red substrate: λ_ex_: 592 nm; λ_em_: 628 nm; cutoff: 630 nm). The cells were kept at 37°C and measured every 30 min for a total of 180 min. Afterward, the cells were washed with PBS and fixed in PBS + 4% w/v formaldehyde (VWR Chemicals, United States, #28794.295) for 20 min at RT. To measure cell volume, the fixed cells were treated with 100 μL of CellTag 700 (LI‐COR Biosciences, United States, # 926–41090) diluted 1:1,000 in blocking buffer (PBS + 0.3% v/v Triton X‐100 + 2% w/v BSA (Sigma Aldrich, United States, #A9647) + 5% v/v FCS) for 1 h RT. Then, all wells were washed three times with PBS, and the plate was scanned in an Odyssey (LI‐COR Biosciences, Lincoln, NE) imaging system.

For data evaluation, PFB‐Gluc measurements over time were normalized per well to the respective CellTag 700 signal (cell volume). Replicates were arranged in an XY diagram with their respective Baf control, and the area under the curve (AUC) for both curves was calculated (Fig. S3). Lysosomal activity was then calculated by subtracting the Baf control (nonlysosomal activity) AUC from the noninhibited AUC (total activity).

### TEM analysis

Fibroblasts were processed for transmission electron microscopy as described previously.^36^, ^37^ In brief, they were grown on plastic support cell culture dishes before fixation and embedding. Thereafter, plastic supports were removed with a shock freezing in liquid nitrogen. After thin sectioning of samples, we transferred them into a JEOL 1400Plus transmission electron microscope (Japan). Images were taken at a nominal magnification of 2000×, and the lysosomal compartment was assessed between patient and control cells.

### Immunofluorescence

Human fibroblasts were cultured on glass cover slides and fixed in 4% v/v formaldehyde prior to immunofluorescent staining. The fixed cells were permeabilized by incubating the slides in PBS + 0.2% saponin (Sigma Aldrich, St. Louis, MO, United States, #47036‐50G‐F) for 5 min, followed by incubation in PBS + 0.2% w/v saponin + 0.12% w/v glycine (AppliChem, Germany, #A1067) for 10 min at RT. The slides were then blocked in blocking solution (PBS + 0.2% w/v saponin + 10% v/v FCS) for 1 h at RT. Anti‐LAMP‐2A rabbit primary antibody, together with anti‐LIMP‐2 goat and anti‐GBA mouse, or anti‐CTSB goat, or anti‐CTSD goat were applied over night at 4°C. All antibodies were diluted 1:100 in blocking solution. After washing the slides three times in PBS, secondary antibodies were diluted 1:500 in blocking solution and applied for 2 h at RT. Slides were washed three times in PBS and stained with DAPI for 10 min. Finally, slides were again washed three times in PBS before mounting on object slides using ProLong™ Gold Antifade Mountant (Thermo Fisher Scientific Inc., #P36930). Imaging was done using a LSM 780 confocal laser scanning microscope (Carl Zeiss, Oberkochen, Germany) using a 40× oil objective (Carl Zeiss, Oberkochen, Germany, #420762‐9900‐000). Co‐localization in the form of Pearson's coefficient was calculated using the Coloc 2 plugin of ImageJ Fiji.^38^ Acquired images were segmented by hand into single cells, and Pearson's coefficient was determined for each cell. Background signal was determined from a control slide that was not incubated with primary antibodies, and the background signal was subtracted from each image before analysis.

**The following antibodies were used for immunofluorescence staining:**

LAMP‐2A Polyclonal antibody (AMC2) (rabbit), Thermo Fisher Scientific Inc./Invitrogen (#51–2200)
LIMP2 Polyclonal Antibody (goat), Thermo Fisher Scientific Inc./Invitrogen (#PA5‐19111)
GBA monoclonal antibody (M01), clone 2E2 (mouse), Abnova (#H00002629‐M01)
Human Cathepsin B antibody (goat), R&D Systems (#AF953)
Human Cathepsin D antibody (goat), R&D Systems (#AF1014)
Donkey anti‐Goat IgG (H + L) Cross‐Adsorbed Secondary Antibody, Alexa Fluor™ 488, ThermoFisher Scientific Inc./Invitrogen (#A11055)
Donkey anti‐Mouse IgG (H + L) Highly Cross‐Adsorbed Secondary Antibody, Alexa Fluor™ 647, ThermoFisher Scientific Inc./Invitrogen (#A‐31571)
Donkey anti‐Rabbit IgG (H + L) Highly Cross‐Adsorbed Secondary Antibody, Alexa Fluor™ 568, ThermoFisher Scientific Inc./Invitrogen (#A10042)

### Homology model and BLAST of GALC protein

The crystal structure of murine GALC (PDB ID 3ZR5^39^) was used for homology modeling with the sequence of human GALC obtained from UniProt^40^ (accession code P54803), created with SWISS‐MODEL (https://swissmodel.expasy.org/). Substrate binding/position was adapted using the murine GALC complexed with 4NBDG (enzyme‐substrate complex; PDB ID 4CCC^41^). Structures were visualized using PyMOL 2.5.5 (DeLano Scientific LLC, Schrödinger Inc., United States). Basic local alignment search tool (BLAST) of UniProt^40^ (https://www.uniprot.org/blast) and the ConSurf Server^42^, ^43^ (https://consurf.tau.ac.il/consurf_index.php) were used to analyze the conservation of certain protein regions.

## Results

### Case report of adulthood‐onset spastic paraplegia

**Figure 2:**
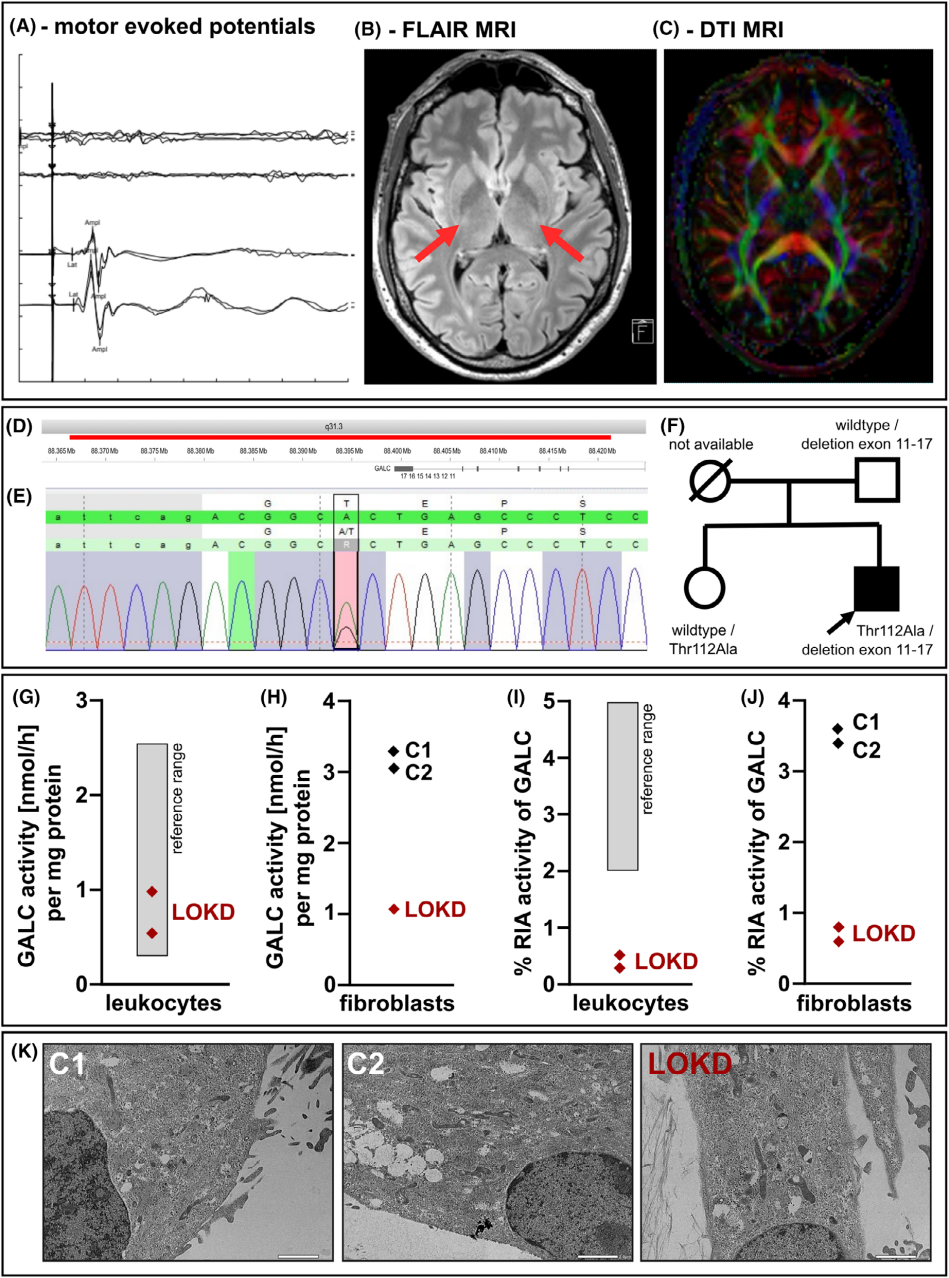
Clinical and genetic characterization of the index patient. (A) Matching the spastic paraplegia phenotype, motor evoked potentials to the Tibialis anterior muscle were bilaterally absent upon motor cortex stimulation (first trace: left motor cortex/right leg, second trace: right motor cortex/left leg, three repetitions each), but unaffected upon lumbar stimulation (third trace: right leg, fourth trace: left leg, two repetitions each). *Vertical division* 2 mV, *horizontal division* 20 ms. (B) Representative image of a fluid attenuated inversion recovery sequence (FLAIR, transversal plane) upon 3.0 Tesla magnetic resonance imaging showing no abnormalities. Specifically, there were no signs of a leukodystrophy and no abnormal hyperintensity of the pyramidal tracts (red arrows). (C) Diffusion tensor imaging (DTI) sequences also showed integrity of all major fiber tracts. (D) Exome sequencing revealed a gene dosage loss of 33 kb (red) spanning exons 11–17 of *GALC*. (E) Moreover, there was a heterozygous c.334A>G variant in *GALC*, leading to a Thr112Ala missense mutation. (F) Segregation analysis was performed both in the father and the sister of the index patient, confirming the compound heterozygous constellation of both, the deletion and the point mutation, variants. (G) In a fluorimetric substrate assay, the GALC activity in the patient leukocyte lysates was not reduced in two repetitive measurements compared to the standard reference range. (H) The same assay applied to fibroblast samples showed a strongly reduced GALC activity in the patient cells compared to two control cultures. Shown is one of three independent time points, all showing the same trend. (I) Utilizing radioimmunoassay (RIA), the relative activity of GALC was reduced (<0.5%) in two repetitive measurements compared to the reference range of healthy controls (>2%). (J) In fibroblast lysates of healthy controls (C1 and C2), GALC activity in RIA was more than three times higher compared to the patient fibroblasts (index case, LOKD). (K) Using transmission electron microscopy (TEM), no changes between control and patient fibroblasts were found regarding the distribution of mitochondria, storage vesicles, or other organelles. No Krabbe bodies were detected. Scale bar: 2 μm.

A 46‐year‐old male patient presented to the movement disorders outpatient unit with a history of gait disturbances for 2 years characterized by increased stumbling along with stiffness of the legs. Family history was unremarkable for neurological diseases. On examination, there was a modest spasticity of the legs, alongside with a symmetrical increase of the lower leg deep tendon reflexes, positive pyramidal tract signs and a mild paresis of the foot elevation. Analysis of cerebrospinal fluid and motor and sensory nerve conduction studies remained unremarkable, but there was a loss of cortical motor evoked potentials to the legs (Fig. 2A). Repeated cerebral magnetic resonance imaging including high field 3.0 Tesla diffusion tensor imaging sequences revealed no unequivocal abnormalities (Fig. 2B,C). Despite absence of myelopathic changes, cervical decompression surgery was performed due the otherwise unremarkable workup and a relative spinal stenosis at C4/5 and C5/6, but symptoms progressed further. At the age of 56 years, maximum gait distance was 1,000 meters using walking sticks, and the affected individual suffered from chronic lower back pain. Due to the progressive spastic paraplegia, exome sequencing was performed. It revealed two variants in the *GALC* gene, both classified as likely pathogenic (Class 4 according to the American College of American College of Medical Genetics^44^), that is, a 33 kb deletion spanning exons 11–17 and the polyA tail of *GALC* (Fig. 2D) and the c.334A>G, p.Thr112Ala, missense variant (further named T112A; Fig. 2E). Carrier testing of the father and the sister of the index patient confirmed the biallelic localization (Fig. 2F). The pathogenicity of the 33 kb deletion is most likely due to a complete loss of the coding information for the 30 kDa subunit and partial loss for the 50 kDa subunit.^45^ The functional consequences of the T112A variant have conflicting interpretations but have mostly been classified as likely pathogenic or with uncertain significance in ClinVar [VCV000092503.54]. Considering the very mild and late‐onset pyramidal syndrome of this patient, we sought to confirm the pathogenicity of the variants *in silico* and *in vitro*, and to characterize other lysosomal components, including the enzyme GCase as potential compensatory mechanism.

### GALC activity is reduced in patient's white blood cells and fibroblasts

Next, we determined GALC enzyme activity in white blood cells and fibroblasts. Using a diagnostically certified fluorometric substrate assay for leukocytes,^29^, ^30^ GALC activity was determined to be 0.54 and 0.98 nmol/h/mg, which are both in the reference range of GALC metabolic rate (Fig. 2G). Using this assay on fibroblast cultures, a lower GALC activity was determined in the patient cells (LOKD) compared to fibroblasts derived from two age‐ and sex‐matched control donors (C1 and C2) (Fig. 2H). To validate these findings, and due to potentially superior sensitivity, GALC activity was additionally measured using the radioactively labeled natural substrate in a radioimmunoassay (RIA).^46^ RIA activity of GALC was pathologically reduced to 0.29 and 0.52% in leukocytes, confirming the diagnosis of KD in this patient (reference range >2%, Fig. 2I). RIA‐GALC activity was also determined in fibroblasts, showing the same trend of reduction in comparison with two controls (Fig. 2J).^30^ These findings provide functional evidence confirming the compound heterozygous variants as likely pathogenic. To identify potential ultrastructural changes in cell and organelle morphology, control and LOKD patient fibroblasts were analyzed by transmission electron microscopy (TEM). Analyzing patient fibroblasts did not reveal obvious morphological or distributional changes in mitochondrial, vesicular, or lysosomal structures (Fig. 2K). Furthermore, there were no indications for Krabbe bodies, which are characteristic intracellular inclusion bodies mostly found in macrophagic cell types of Krabbe disease patients.^47^

### Missense mutation in highly conserved region of GALC

**Figure 3:**
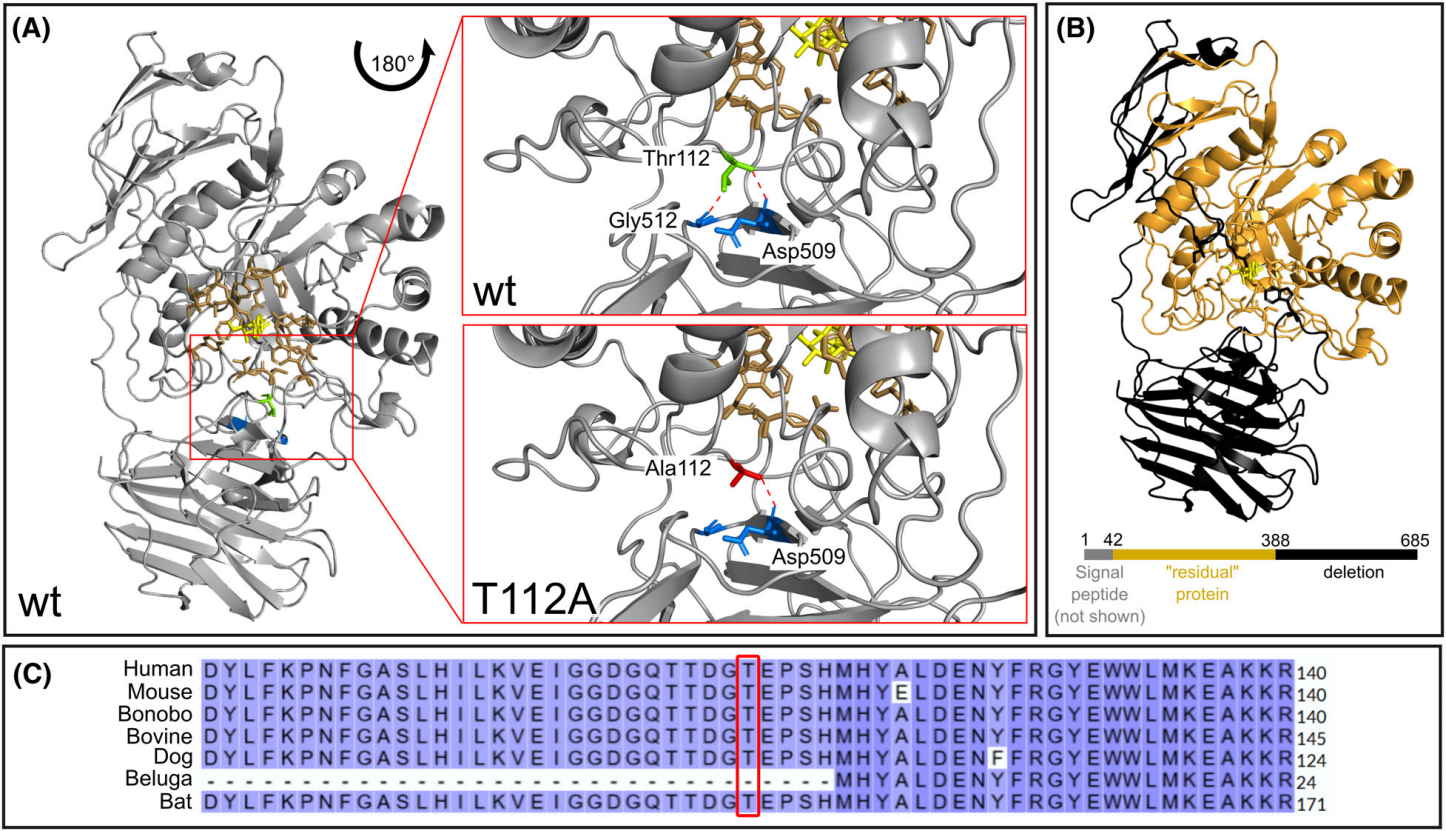
Homology modeling of GALC wt, T112A, and the deletion variant. (A) Homology modeling of human GALC wt (gray, based on PDB file 3ZR5^39^) with bound substrate (yellow, implemented from PDB file 4CCC^41^). Thr112 (green) is in close distance to the residues interacting with the substrate (brown), yet not directly involved in substrate binding. Thr112 builds hydrogen bonds with Asp509 and Gly512 (red dashed lines, wt/top), while the variant with Ala112 (red) seems to lose at least one hydrogen bond to Gly512 (red dashed line, T112/bottom). Glycosylation sides are not shown. (B) The deletion variant of *GALC* lacks residues originated from exon 11 to 17. In consequence, a large part of the protein, including the substrate interaction side/binding pocket, is missing (black); thus, the functionality of the remaining protein (orange) is unlikely. (C) BLAST alignment (https://www.uniprot.org/blast) of GALC residues around Thr112 between human and six other mammalians. Except for GALC of species like Beluga whale, which has over 100 residues less than human GALC, the region around Thr112 and is highly conserved within species.

To understand the structural impact of the *GALC* p.Thr112Ala missense variant, we used 3D simulations and homology modeling to investigate a possible impact on the protein's structure, potentially affecting its stability or activity. The first 24 amino acids of GALC encode the signal peptide, which is not solved in the crystal structure and not part of the mature enzyme as presented here. The signal peptide is followed by three main protein domains: a triosephosphate isomerase barrel (residue 41–337), a β‐sandwich domain (residues 338–452), and a lectin domain (residues 372–668).^39^ The residues mostly interacting with the substrate are Thr109, Trp151, Asn197, Glu198, Tyr254, Ser277, Glu274, Trp307, and Tyr319^39^ (Fig. 3A, brown residues). Interestingly, the main binding surface is formed by the triosephosphate isomerase barrel, but also both other domains contribute to substrate binding. However, the residue of the missense variant T112A described here is located close to the substrate‐binding interface, but it is not directly involved in substrate binding. The wildtype (wt) protein harboring a threonine at position 112 interacts with neighboring residues Gly512 and Asp509 (Fig. 3A, wt/top). Insertion of an alanine at this position leads at least to loss of a hydrogen bond with Gly512 (Fig. 3A, T112A/bottom). On the other hand, the deletion variant of *GALC* causes a loss of huge protein parts, which makes correct and functional folding of the residual protein and the binding surface for substrates very unlikely^48^ (Fig. 3B). BLAST and conservation score analysis show a high conservation of GALC and the residues around Thr112, especially among mammals (Fig. 3C). In comparison of 150 sequences, Thr112 is at conservation score seven out of nine (Fig. S1), suggesting that the replacement of Thr is more likely to have effects on GALC function and stability compared to less conserved/more variable residues.

### Lysosomal protein expression in LOKD

**Figure 4:**
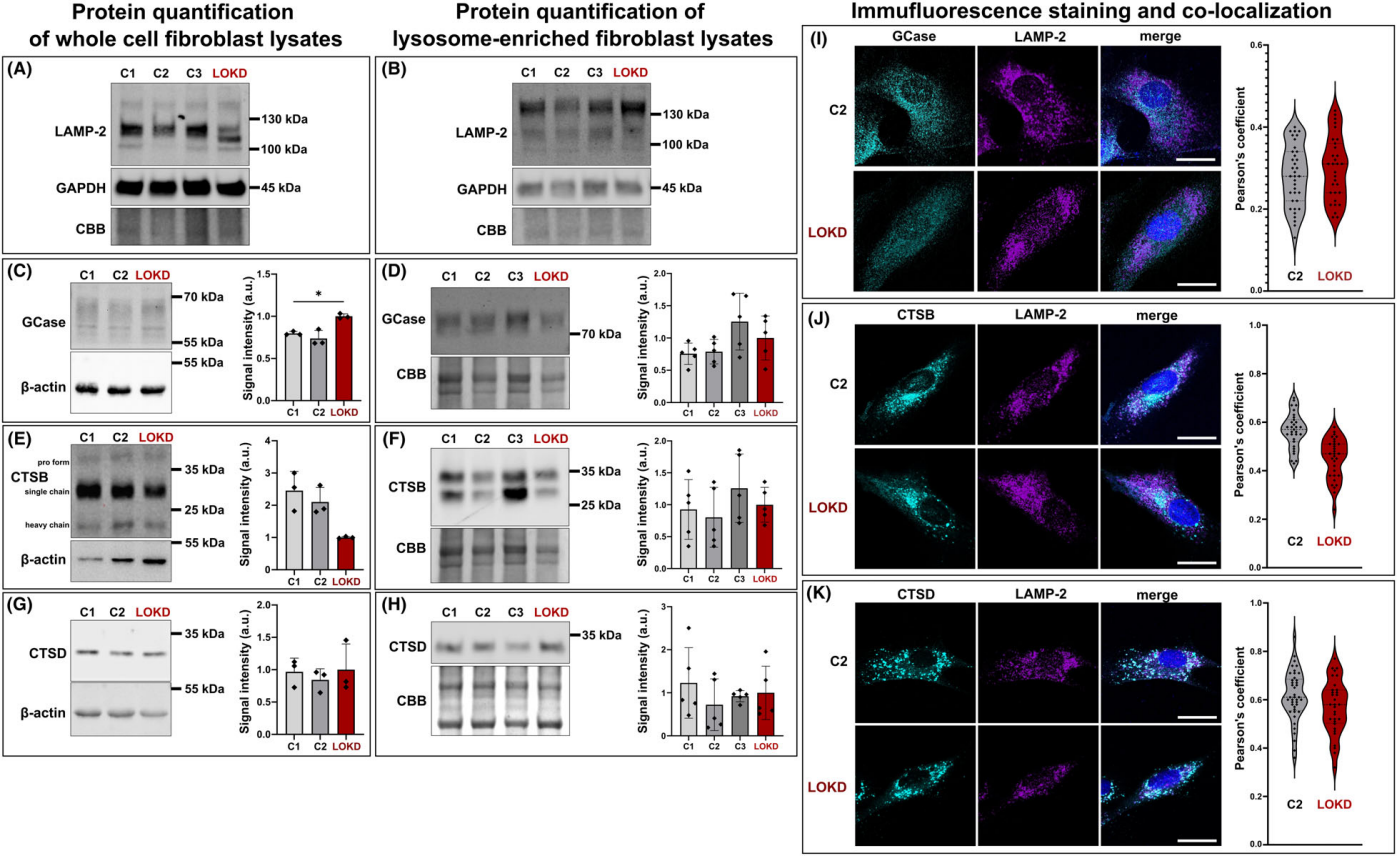
Protein levels of lysosomal proteins in lysates of control and patient fibroblasts. (A and B) Comparison of protein expression patterns of LAMP‐2 in whole fibroblast lysates (A) and lysosome‐enriched fibroblast lysates (B). In lysosomes, LAMP‐2 is in a more glycosylated state. (C–H) Western blots and signal intensities of lysosomal enzymes: GCase (C and D), CTSB (E and F), and CTSD (G and H) expression levels. C, E, and G display protein levels from whole‐cell fibroblast lysates, normalized to β‐actin, with an *n* = 3 of two control (C1 and C2) and the patient (LOKD) fibroblast lines. GCase levels were significantly higher for LOKD in comparison with C1 in whole‐cell lysates (C). CTSB and CTSD expressed at similar levels in control and LOKD (E and G). D, F, and H display protein levels from lysosome‐enriched fibroblast lysates, normalized to total protein load (Coomassie Brilliant Blue (CBB)) with an *n* = 5 of three controls (C1–C3) and patient line (LOKD). All three proteins have comparable levels between all controls and LOKD. Statistics: Welch's ANOVA test with Dunnett's T3 multiple comparison: *p < 0.05. (I–K) Immunofluorescence staining of control (C2) and LOKD fibroblast utilizing LAMP‐2 (purple) as lysosomal marker, and correlating to different lysosomal enzymes (GCase (I), CTSB (J), or CTSD (K); turquois). Shown are representative pictures. No obvious alterations in location or distribution could be seen, and the Pearson's coefficient was similar between C2 and LOKD (*n* = 30–40). Blue: nucleus (DAPI); scale bar: 25 μm.

To analyze whether protein levels of lysosomal enzymes are altered due to GALC malfunction, western blot analyses were performed from lysates as well as lysosome‐enriched fractions of patient and control fibroblasts (Fig. 4). To evaluate the quality of our lysosomal enrichment, the signal of the lysosome‐associated membrane protein‐2 (LAMP‐2/CD107b) was compared between whole‐cell and lysosome‐enriched lysates. Differences in expression pattern of LAMP‐2 between both samples indicate the presence of higher matured and glycosylated forms of the protein in the lysosomal fraction (Fig. 4A,B), suggesting successful lysosomal enrichment. Following, the protein levels of further lysosomal enzymes were analyzed in both sample preparations (whole lysate (Fig. 4, left panel: C, E, G) and lysosome‐enriched lysate (Fig. 4, right panel: D, F, H)) in LOKD in comparison with control samples: GCase (Fig. 4C,D), CTSB (Fig. 4E,F), and CTSD (Fig. 4G,H). Although we found a significant increase in GCase protein expression in whole‐cell lysate of LOKD compared to one control (Fig. 4C), we could not detect other significant alterations of GCase, CTSB, or CTSD in neither whole‐cell nor lysosome‐enriched lysates. Additionally, we also analyzed the autophagy receptor sequestosome 1 (SQSTM‐1/ubiquitin‐binding protein p62), which is involved in the cellular stress response^49^ (Fig. S2A), and another lysosomal membrane protein, the lysosome integral membrane protein type‐2 (LIMP‐2), that is also the lysosomal transporter of GCase^50^ in whole‐cell lysates (Fig. S2B). For both proteins, no significant changes were observed in LOKD in comparison with controls. In order to visualize the lysosomal localization of the enzymes, we performed immunofluorescence staining of GCase (Fig. 4I), CTSB (Fig. 4J), and CTSD (Fig. 4K) in co‐staining with the lysosomal marker protein LAMP‐2. Co‐localization analyses via Pearson's coefficient did not exhibit differences in the lysosomal localization of the three enzymes. Further, LAMP‐2 signals within the cells indicate no obvious differences regarding size, volume, and distribution of lysosomes in the LOKD patient in comparison with the control (Fig. 4I–K). Also, LIMP‐2 signal in co‐staining with GCase did not indicate differences of co‐localization and an obvious change in lysosomal morphology in LOKD cells (Fig. S2C).

### Changes in lysosomal enzyme function in LOKD

**Figure 5:**
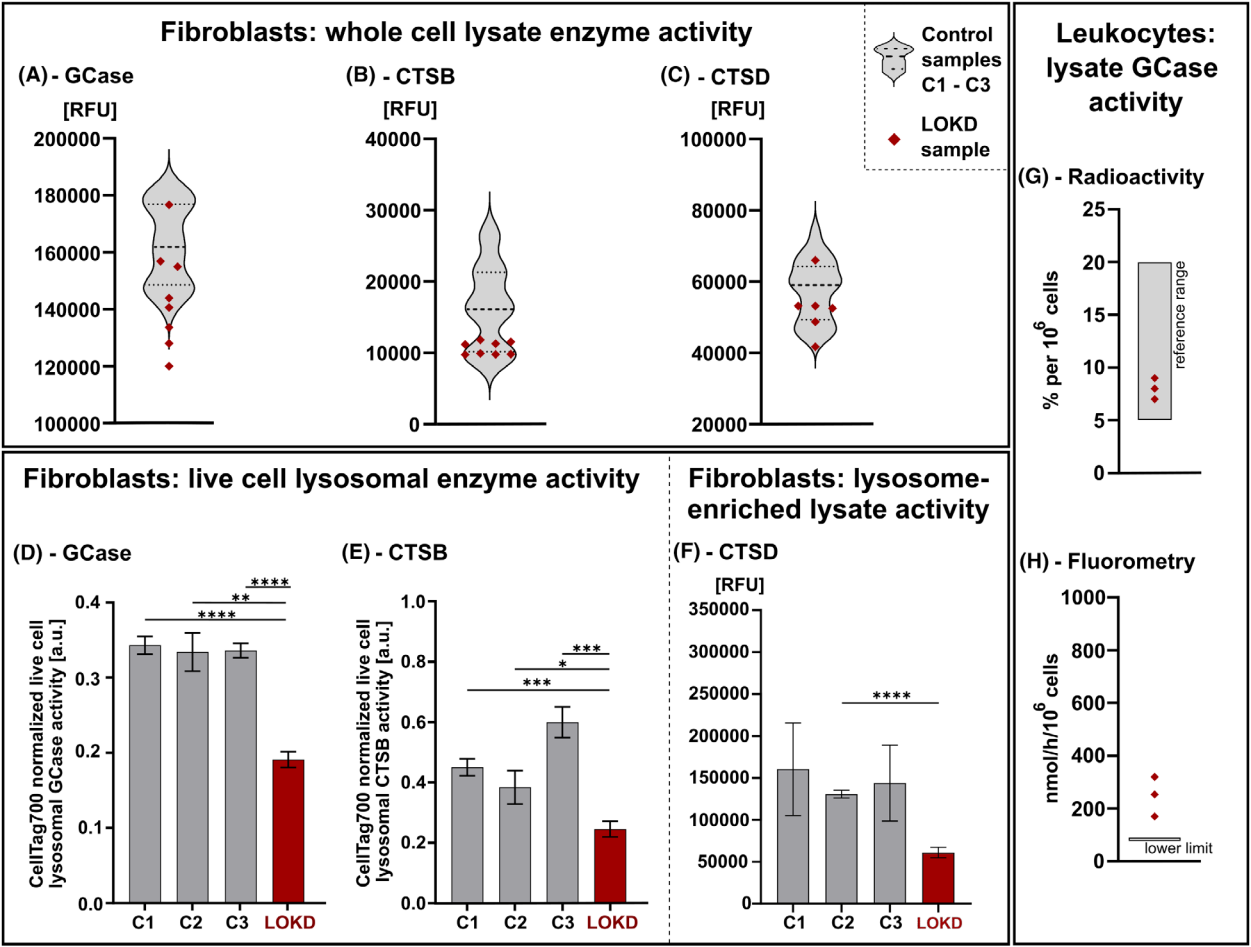
Analyses of lysosomal enzyme activities in fibroblasts and leukocytes. (A–C) Activities of GCase (A), CTSB (B), and CTSD (C) were determined in lysates of fibroblasts using artificial fluorogenic substrates. The activity of all measured enzymes was ranging in the same level in LOKD fibroblast (red dots) in comparison with all control samples (C1–C3, gray violin plot, *n* = 6–8). (D and E), Lysosomal enzyme activities of GCase (D) and CTSB (E) were measured in live fibroblasts by an established assays utilizing cell membrane‐permeable fluorogenic substrates and normalization to cell volume (CellTag700 signal). Raw values of lysosomal activities of individual samples can be found in Figure S3. The enzyme activity of both proteins was significantly reduced in the LOKD samples (*n* = 4). (F) Activity of CTSD in lysosome‐enriched fibroblast lysates (*n* = 4), determined using artificial fluorogenic substrate. Statistics: Welch's ANOVA test with Dunnett's T3 multiple comparison: ****p < 0.0001, ***p < 0.001, **p < 0.01, *p < 0.05. (G and H) Degradation capacity of glucocerebroside in LOKD leukocytes determined by radioactivity analysis was within the reference range (*n* = 3, G). Fluorometry analysis revealed values above the lower boundary value of 80 nmol/h/10^6^ cells (*n* = 3, H).

To correlate protein expression and enzymatic function, we analyzed activity of the lysosomal hydrolases GCase, CTSB, and CTSD that all have been linked to neurodegeneration, including PD.^24^, ^25^ First, we used fluorogenic substrate assays in fibroblast lysates (Fig. 5A–C). Values of the LOKD index patient were in range of three control samples for all three enzymes, yet with a tendency to lower activity in GCase and CTSB (Fig. 5A,B). To overcome the limits of whole‐cell lysate analyses, GCase and CTSB activity were additionally determined specifically in lysosomes of live fibroblasts^35^ (Fig. 5D,E). In this context, we measured significantly reduced live cell lysosomal activities of both enzymes (GCase and CTSB) in the LOKD cells compared to each control. As the artificial fluorescent substrate to determine the enzymatic activity of CTSD is not membrane‐permeable, we analyzed the lysosomal CTSD activity in lysosome‐enriched fractions (Fig. 5F). We found that the LOKD sample showed reduced CTSD activity, however only toward one control line (C2) with high significance. Reduced lysosomal activities of key and disease‐associated lysosomal enzymes like GCase, CTSB, and CTSD in the presence of impaired GALC activity indicate functional interdependence and underline converging disease mechanisms between lysosomal dysfunction and neurodegeneration. Additionally, we measured the degradation capacity of GCase in the LOKD patient's leukocyte lysates using radioactivity (Fig. 5G) and an artificial fluorescent substrate (Fig. 5H). Although GCase activity was not significantly decreased, it was on the low side of the reference range for both assays.

## Discussion

In the present report, we demonstrate a comprehensive approach validating the pathogenicity of the genotype in a patient with a late‐onset spastic paraplegia phenotype. Besides in‐depth clinical and neuroradiological phenotyping, we established functional evidence to confirm the pathogenicity based on *in vitro* GALC enzyme activity and a histopathological characterization of the lysosomal function. We uncovered the compromised lysosomal activity of GCase, CTSB, and CTSD in live cell fibroblast lysosomes (Fig. 5‐DF) that may further contribute to a lysosomal dysregulation. Noteworthy, no decreased activity was observed in leukocyte homogenates, thus highlighting the importance of analyzing in particular the lysosomal activity of different enzymes besides GALC in live cell assays in KD studies. Determination of GALC activity levels from fibroblasts instead of leukocytes (Fig. 2H,J) showed promising, potentially more robust differentiation between KD and control cells, thus, these methods might be considered for future studies.

As large C‐terminal deletions in the *GALC* gene cause a loss of enzyme activity,^45^ the reduced GALC activity shown in Figure 2G–J most likely represents residual activity of the c.334A>G missense allele. A previous functional GALC expression study conducted on this variant using COS1 cells observed reduced GALC activity compared to wt‐GALC, with GALC activity even more significantly reduced when an additional *GALC* missense variant was concurrently present.^51^ Noteworthy, a report of an early‐onset case carrying the homozygous c.334A>G variant led to the conclusion that this variant is sufficient for KD development.^52^ All other cases describe the c.334A>G variant as insufficient in provoking KD alone and LOKD cases develop only in the presence of a second pathogenic variant.^51^, ^52^, ^53^ Four homozygous carriers (0.25%) are present in GnomAD [14‐88452941‐T‐C, accessed 25Mar2024], but it is well possible that some of these were in the presymptomatic stage of KD. As described, we found reduced GALC activity in the presented patient who carried one loss of function variant, leaving the c.334A>G variant responsible for all residual GALC activity. The clinical constellation in the presented index patient with a relatively late and mild KD progression further supports the notion that homozygous c.334A>G variant causes mild GALC impairment, but is not sufficient to induce an early‐onset KD phenotype.

The high conservation of Thr112 in GALC and nearby residues among different species suggests their importance for protein function. Consistently, missense variants c.331G>A (p.Gly111Ser), c.332G>A (p.Gly111Asp), and c.349A>G (p.Met117Val) close by are listed as (likely) pathogenic (VCV001163373.7, VCV000211058.17 and VCV000092504.13). The loss of at least one hydrogen bond and potentially other interaction sites between Thr112 and its surrounding structure could impair protein stability and affect the interaction with substrates, that is, by increased flexibility or conformational changes in the substrate‐binding area. Furthermore, the surface charge of proteins is pH dependent, thus stability and flexibility of GALC depend on the environment. Amino acids such as aspartic acid, glutamic acid, and histidine may undergo changes in their protonation state depending on the pH level,^54^ which can also affect the glutamic acids involved in substrate binding (Glu198 and Glu274). The ConSurf estimation suggests several residues in close proximity to Thr112 as highly conserved and essential for the protein structure (Fig. S1). For lysosomal proteins with highest activities at low pH, alterations on the protein surface – especially near the substrate‐binding surface – can potentially cause stronger effects when translocated to the lysosome.^39^

LOKD patients can lack the typical hallmarks in magnetic resonance imaging, like cortical atrophy and white matter changes,^55^ which makes reliable tests of enzymatic activity more important in order to confirm pathogenicity of *GALC* variants. Albeit we did not perform a direct comparison of both methods of GALC enzyme determination, our findings support the notion that RIA assays (Fig. 2I,J) might have a superior sensitivity to test GALC enzymatic activity in comparison with artificial substrate testing,^46^ which had shown activity levels within the normal range (Fig. 2G). This is due to conventional fluorogenic substrates lacking specificity toward GALC and producing unspecific background signal via cleavage through β‐galactosidases.^56^ Furthermore, fibroblasts might provide a more powerful resource when testing for enzymatic activity of lysosomal enzymes than leukocytes (Fig. 2H,J). Nevertheless, additional systematic analyses of KD patients and controls are necessary to evaluate both (fibroblast and leukocyte) assays' exact sensitivity and specificity. Additionally, determination of psychosine levels could be an additional way to test for pathogenicity of *GALC* variants^57^ for early‐onset Krabbe disease; however, data in late‐onset Krabbe cases are sparse. As disease severity has been shown to be linked to the cellular localization of the GALC enzyme,^58^ analysis of GALC activity in lysosomes might deliver further information of disease state and progression. Preliminary measurements of GALC activity in lysosome‐enriched fractions of fibroblasts showed a decrease of the residual activity in LOKD comparable to the enzyme activity measured in leukocytes (data not shown).

Beside reductions in the GALC activity, we also observed a decrease in lysosomal GCase, CTSB, and CTSD activity in patient fibroblasts. Impaired GALC function might affect protein expression, maturation, and/or activity of other enzymes as an alternative way to compensate the lack of degradation products (i.e., ceramides, see Fig. 1). Protein and activity levels do not necessarily correlate with each other, as GCase expression was increased in LOKD whole‐cell lysates, yet not in lysosome‐enriched fractions (Fig. 4C,D), although activity levels were significantly reduced in lysosomes (Fig. 5D). Similarly, no altered protein levels of CTSB and CTSD could be found in whole‐cell or lysosome‐enriched lysates (Fig. 4E–H); however, both revealed significantly reduced activity levels in LOKD fibroblasts (Fig. 5E,F).

We hypothesize that alterations in GCase, CTSB, and CTSD activity might be caused via pathway interactions of the reduced GALC activity, in particular via reduced ceramide levels. While GCase and GALC are essential players in ceramide metabolism, CTSB and CTSD are interaction partners of ceramides, for example, being altered by ceramides in their maturation, biogenesis, transport, and activity.^59^, ^60^, ^61^, ^62^, ^63^ A reduced CTSB activity has been seen in the LSD Niemann–Pick type C1 and C2^64^ (NPC1 and 2), which is caused by dysfunction of cholesterol transporters 1 and 2, leading to an impairment in the metabolization of cholesterol and sphingomyelin^65^, ^66^ (Fig. 1). Interestingly, sphingomyelin has been reported to inhibit cholesterol transfer in NPC2, which points to an important role of acid sphingomyelinase (ASM) for cholesterol exit.^67^ Deficiency in ASM activity, important for the processing of sphingomyelin to ceramide (Fig. 1), is related to Niemann–Pick type A and B (NPA and NPB),^68^ and genetic variants have been associated with PD.^26^, ^69^, ^70^ Further, CTSB and CTSL deficiency was shown to cause NPC‐like cholesterol sequestration.^71^

**Figure 6:**
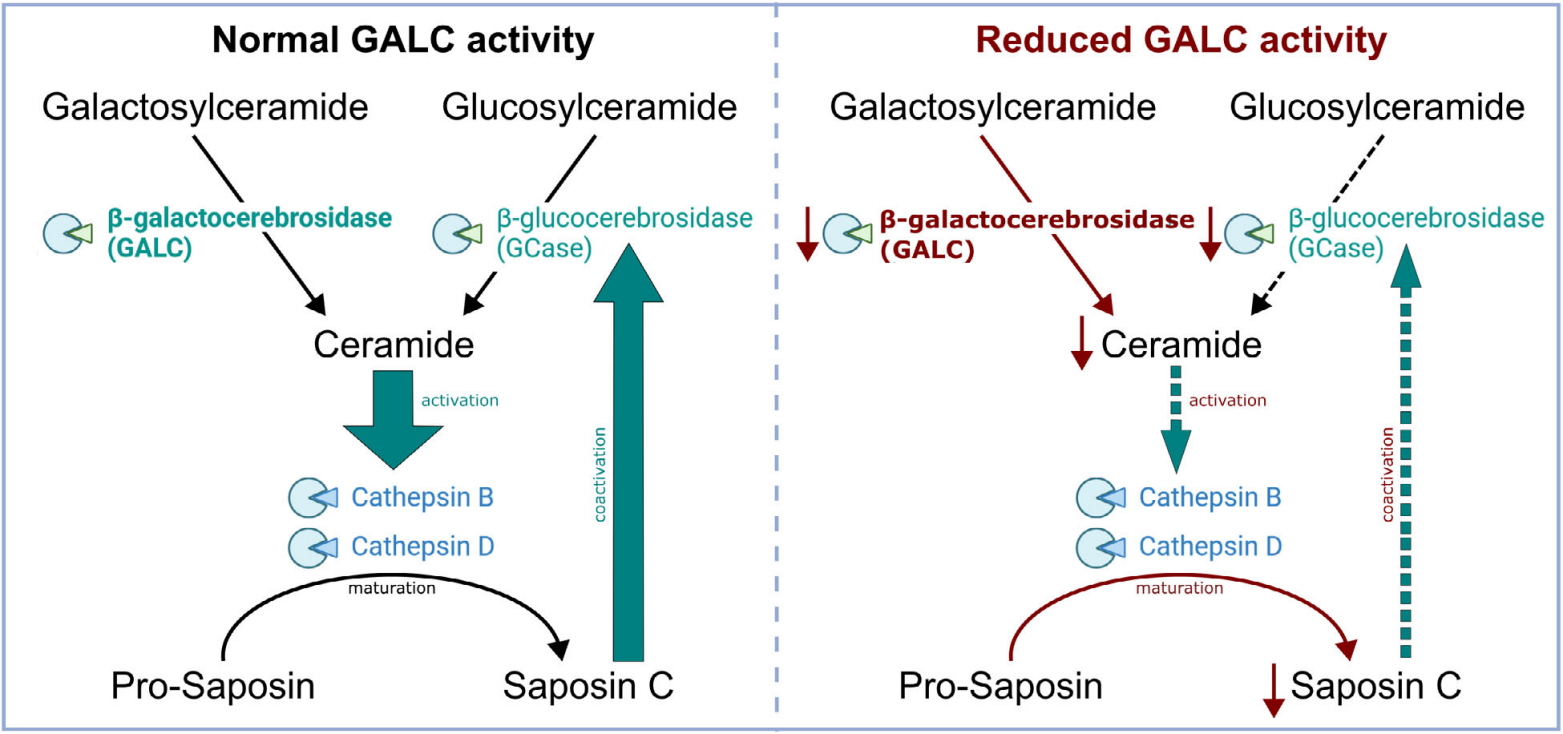
Overview of interaction and interdependence of GALC with other lysosomal enzymes and substrates. Reduced activity of GCase, CTSB, and CTSD – as seen in our study – can be correlated to ceramide levels: Fully functional (left) GALC produces, together with other enzymes, sufficient amounts of ceramides, which is essential for appropriate activation or maturation of CTSB and CTSD. These subsequently promote maturation of pro‐saposin to saposin C, which serves as a co‐activator for GCase. In consequence, reduced GALC activity (right) as in our LOKD patient leads to reduced ceramide levels. Less CTSB and CTSD is activated and pro‐saposin remains in its non‐maturated state. Missing the co‐activation, GCase activity is reduced and, as already caused by malfunctioning GALC, ceramide levels decrease even further. *Figure created with Inkscape and BioRender.com*.

The here observed changes in lysosomal GCase, CTSD, and CTSB activity might be consequence of a decreased maturation rate of pro‐saposin to saposin C, which an essential co‐activator of GCase,^60^ as outlined in Figure 6. CTSB and CTSD are able to cleave pro‐saposin into the mature homologs including saposin C, potentially leading to the lack of saposin C‐mediated GCase co‐activation.^60^, ^72^ Overall, this may result into a vicious cycle: a GALC‐dependent decrease in ceramide results in less active CTSB and CTSD, which leads to a reduced cathepsin‐dependent maturation of saposin C. Consequently, a reduction of the saposin C level is accompanied by reduced GCase activity, feeding into diminished ceramide production, as already being present due to GALC deficiency (Fig. 6). Our study supports this hypothesis as we saw significantly reduced lysosomal activities of GCase, CTSB, and CTSD next to the present GALC deficiency, thus indicating a relevant interconnection of lysosomal enzymes and their substrates. Measurement of intermediates and substrates in future studies should corroborate here described interactions.

Importantly, several variants in *GBA1*, *CTSB*, and *CTSD* are either causal for PD or genetic risk factors for PD.^21^, ^23^, ^73^ Remarkably, single nucleotide polymorphisms affecting *GALC* gene expression have also been associated with increased PD risk.^23^, ^26^, ^74^ At least one *GALC* variant with enhanced protein expression levels and activity has been associated to PD^27^. In contrast, *GALC* knockout in human pluripotent stem cell derived neurons did neither affect GCase activity, nor induce accumulation of α‐synuclein. It has to be mentioned that in this case GCase activity was determined from whole‐cell lysate and not in the lysosomal compartment. Moreover, only soluble α‐synuclein was determined in this study,^27^ lacking the analysis of insoluble, disease‐associated α‐synuclein conformers. Using the Twitcher mouse model, carrying a single mutation abrogating GALC activity, α‐synuclein inclusions could be detected in brain regions correlated with early symptoms of KD, as well as in the brain of three infantile KD cases.^75^ The accumulation of α‐synuclein over longer time periods can potentially be linked to LOKD and PD, especially as the variety of symptoms cannot easily be correlated with one or the other. Hence, biochemical, functional, and genetic analyses of *GALC* in PD patients are needed, to validate potential correlations between the molecular mechanisms of PD and LOKD. Also, more in‐depth biochemical analyses of pathology‐associated α‐synuclein conformers, which are a hallmark of PD – either by structure‐specific antibodies or via the analysis of insoluble α‐synuclein fractions – should support a more complete picture. As α‐synuclein accumulation has been shown to mediate a negative effect on the lysosomal trafficking of GCase, CTSB, and CSTD,^76^, ^77^ further triggering α‐synuclein accumulation,^77^, ^78^, ^79^ it would be interesting to study this reciprocal interaction also in a *GALC*‐deficient background. However, as fibroblasts do not express α‐synuclein, the precise interplay of α‐synuclein and GALC dysfunction needs to be established in another cell system than utilized in this study.

Overall, the present report highlights the difficulties in diagnosing late‐onset KD and the importance of performing RIA analysis of GALC activity in leukocytes or even fibroblasts. Moreover, our data identify alterations of additional lysosomal enzymes (GCase, CTSB, and CTSD) that may also be implicated in KD pathogenesis by reduced functionality within the ceramide cycle, where maturation of cathepsins and subsequent cofactors, like saposin C, are essential to keep the ceramide metabolism in a physiological state. We recommend to include protein expression and activity analyses of lysosomal enzymes in future KD/GALC studies to find more evidence about cross‐interactions in lysosome metabolism.

## Author Contributions

R.M., M.R., and F.Z. wrote the manuscript and provided figures. R.M. J.D., A.W., and M.K. performed experiments. R.M., J.D., M.K., S.G., and L.L. performed data analysis. M.S. performed magnetic resonance imaging and analysis. U.H. performed exome analysis. R.M. and P.A. performed homology/structure modeling. P.A. performed TEM analysis. M.R. and F.Z. supervised the study and provided funding. All authors were involved in proof reading and preparation of the final version of the manuscript.

## Conflict of Interest

The authors declare that they have no known competing financial interests or personal relationships that could have appeared to influence the work reported in this paper.

## Supporting information


Figure S1.

Figure S2.

Figure S3.

## Data Availability

For data protection reason, patient exome data may not be made public; all other data sources are listed in the methods.
