# Supplementary figures and images for: Late‐onset Krabbe disease presenting as spastic paraplegia – implications of GCase and CTSB/D
Source: Ann Clin Transl Neurol. 2024 Jun 4;11(7):1715–31. doi: 10.1002/acn3.52078 (PMC11251474; doi:10.1002/acn3.52078)

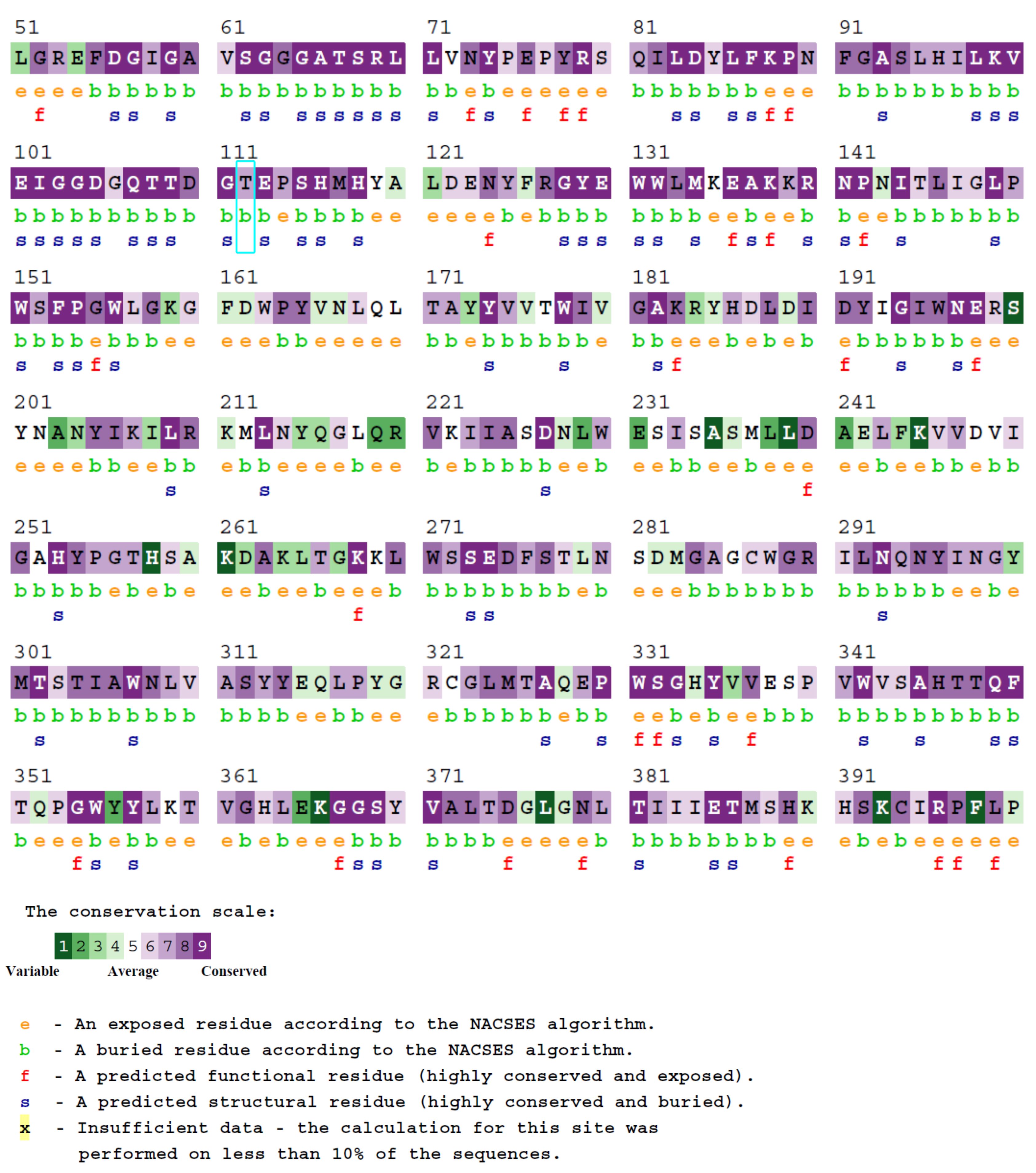

Supplement: Supplementary file 1 — Figure S1. [file ACN3-11-1715-s001.png]

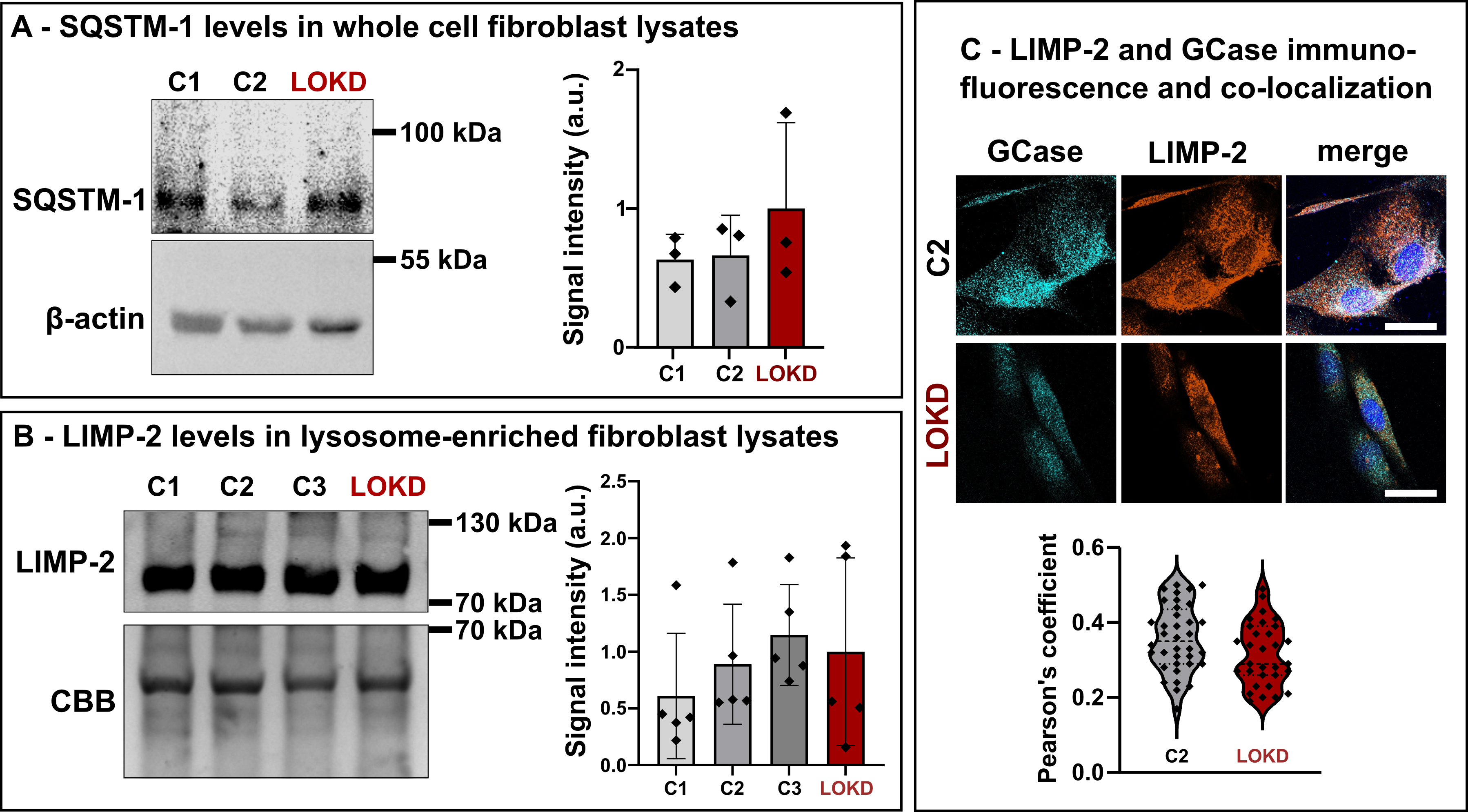

Supplement: Supplementary file 2 — Figure S2. [file ACN3-11-1715-s002.png]

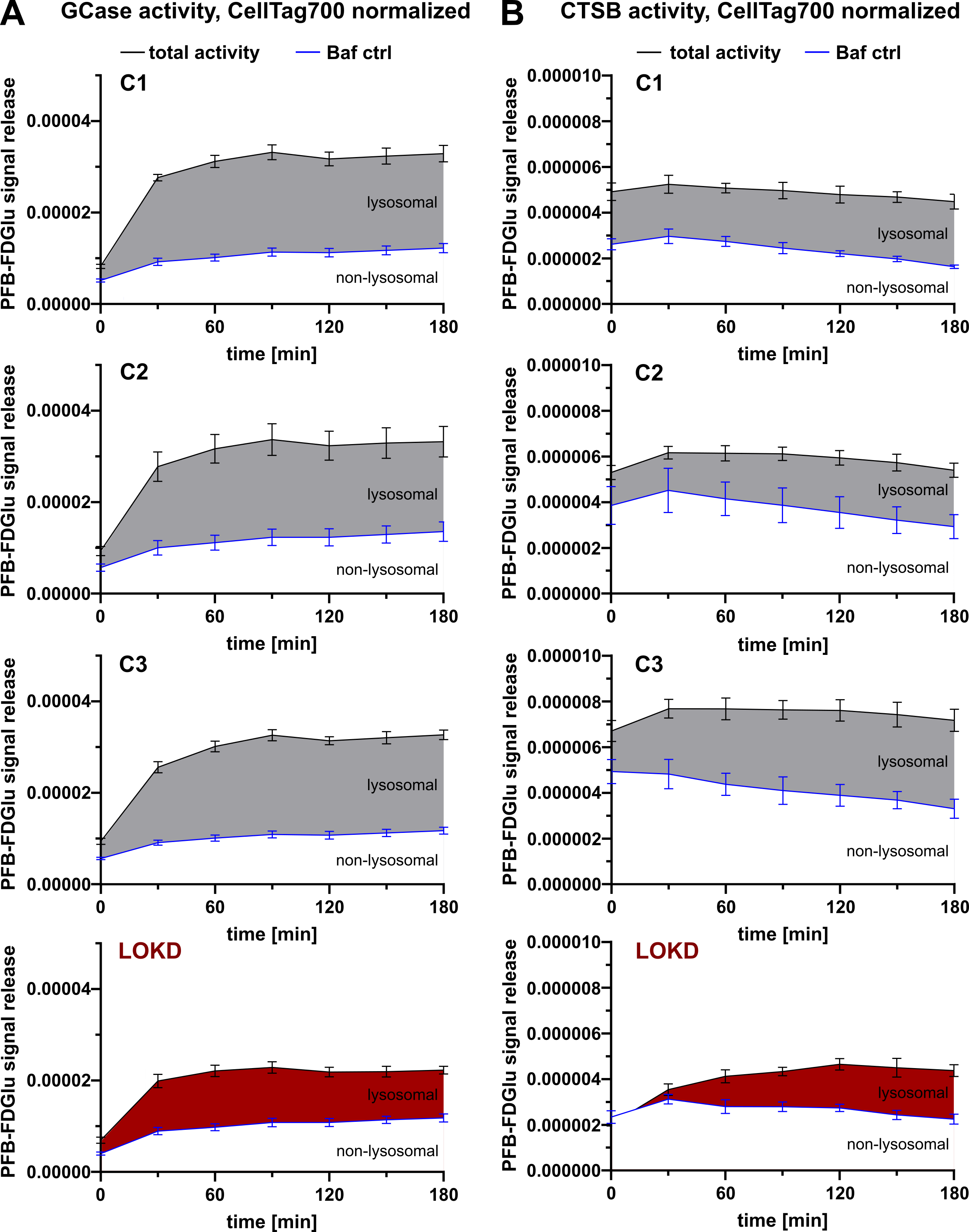

Supplement: Supplementary file 3 — Figure S3. [file ACN3-11-1715-s003.png]
